# Lung development in laminin γ2 deficiency: abnormal tracheal hemidesmosomes with normal branching morphogenesis and epithelial differentiation

**DOI:** 10.1186/1465-9921-7-28

**Published:** 2006-02-16

**Authors:** Nguyet M Nguyen, Leena Pulkkinen, Jessica A Schlueter, Guerrino Meneguzzi, Jouni Uitto, Robert M Senior

**Affiliations:** 1 Department of Internal Medicine, Washington University School of Medicine, St. Louis, Missouri, USA; 2 Department of Cell Biology & Physiology, Washington University School of Medicine, St. Louis, Missouri, USA; 3 Department of Clinical Nutrition, University of Kuopio, Kuopio, Finland; 4 INSERM U634, School of Medicine, University of Nice-Sophia Antipolis, Nice, France; 5 Department of Dermatology and Cutaneous Biology, Jefferson Medical College, Thomas Jefferson University, Philadelphia, Pennsylvania, USA; 6 Department of Biochemistry and Molecular Biology, Jefferson Medical College, Thomas Jefferson University, Philadelphia, Pennsylvania, USA

## Abstract

**Background:**

Laminin γ2 (*Lamc2*), one of the polypeptides in laminin-332 (laminin-5), is prominent in the basement membrane of alveolar walls and airways of developing and adult lung. Laminins are important for lung morphogenesis and based on its localization, a function for laminin γ2 in lung development has been hypothesized. Targeted deletion of the laminin γ2 gene in mice results in skin blistering and neonatal death at 3–5 days after birth due to failure to thrive.

**Methods:**

Examination of lung development in *Lamc2-/- *mice through 1–2 days postnatal was accomplished by morphometric analysis, lung bud culture, electron microscopy, immunohistochemical and immunofluorescence staining.

**Results:**

Compared to littermate controls, *Lamc2-/- *lungs were similar in morphology during embryonic life. At post-natal day 1–2, distal saccules were mildly dilated by chord length measurements. Epithelial differentiation as evaluated by immunohistochemical staining for markers of ciliated cells, Clara cells, alveolar type I cells and alveolar type II cells did not reveal a difference between *Lamc2-/- *and littermate control lungs. Likewise, vascular development, smooth muscle cell differentiation, and elastic fiber formation looked similar, as did airway basement membrane ultrastructure. Branching morphogenesis by lung bud culture was similar in *Lamc2-/- *and littermate control lungs. Since laminin-332 is important for hemidesmosome formation, we examined the structure of tracheal hemidesmosomes by transmission electron microscopy. Compared to littermate controls, *Lamc2-/- *tracheal hemidesmosomes were less organized and lacked the increased electron density associated with the basement membrane abutting the hemidesmosome.

**Conclusion:**

These findings indicate that laminin γ2 and laminin-332, despite their prominence in the lung, have a minimal role in lung development through the saccular stage.

## Background

Lung morphogenesis requires coordinated input from a multitude of diverse molecules ranging from transcription factors to growth factors to cytokines and extracellular matrix. Basement membranes are specialized extracellular matrices that have vital roles in cell adhesion, migration, differentiation, as well as in tissue organization and development [1]. Laminins, type IV collagen, entactin/nidogen, and sulfated proteoglycans are the main components of basement membranes. Laminins are heterotrimers composed of one α, one β, and one γ chain. To date, 5α chains, 4β chains, and 3γ chains are present in humans and mice and these laminin chains self-assemble to form at least 15 laminins [2].

The lung is rich in laminin chains and all but the laminin γ3 chain (which is not present in lung basement membrane [3]) are detected in the lung at some point during development and in adult lungs. The laminin α1-α5, β1-β3, and γ1-γ2 chains are present in embryonic lung; laminin α2-α5, β1-β3, and γ1-γ2 chains are present in the adult lung [4-7]. Studies of lung development from our laboratory and others have shown that laminin and its interactions are crucial for lung morphogenesis. Epithelial-derived laminin chains are important for lung development since addition of either laminin-111 (formerly laminin-1) antibodies or proteolytic fragments to lung bud cultures perturbs branching morphogenesis [8]. Interference with entactin/nidogen binding to laminin through ablation of the nidogen-binding site on laminin γ1 in vivo affects sacculation [9]. Mesenchymal cell-derived laminin α2 is required for bronchial smooth muscle cell differentiation in vitro [10]. Targeted deletion of laminin α5 in the mouse results in abnormal lobar septation, absence of visceral pleura basement membrane, and ectopic deposition of laminin α4 in lungs through embryonic day 16.5 at which time these mice die [11]. Ablation of laminin α5 expression by lung epithelial cells alone via the SP-C promoter and the Cre/LoxP system enabled examination of lungs up to post-natal day 1. The lungs had grossly enlarged distal airspaces and markedly impaired distal epithelial cell differentiation [11]. Thus, multiple in vitro and in vivo studies have shown that laminins are important for lung development at different stages.

Laminin γ2 which is unique to laminin-332 (formerly laminin-5), localizes to airway epithelial basement membranes during lung development leading to speculation that it is required for lung development [12-14]. Laminin γ2 null *(Lamc2-/-) *mice exhibit blistering and erosions of the skin and die a few days after birth presumably due to malnutrition as a result of blistering and erosions in the oral cavity [15], but the lungs have not been described. In this investigation, we report findings regarding lung development in *Lamc2-/- *mice.

## Methods

### Production, breeding and genotyping of mice

Production of *Lamc2-/- *mice has been described previously in detail [15]. *Lamc2-/- *mice were maintained on a mixed 129/C57BL/6J background. Timed matings of mice were established to produce *Lamc2-/- *and littermate control offspring at various ages. Noon on the day of detection of a vaginal plug was designated as E0.5. Genomic DNA was obtained from tails using the Qiagen DNA kit (Qiagen, Carlsbad CA). Genotyping was completed by PCR with laminin γ2 specific primers, wild type forward 5'-CCG CTT GCT GAC TTG TAT CC-3', *Lamc2-/- *forward 5'-AGC TAA TAC GGG TTC AGC C-3', reverse 5'-TGT AAC CAG AAG CAC ATT CC-3'. The Washington University Animal Studies Committee approved all experiments.

### Antibodies

Rat monoclonal antibody to murine laminin α1 was from Dale Abrahamson (University of Kansas Medical Center). Rabbit polyclonal antibodies to laminin α3A and α3B were from Takako Sasaki (Max Planck Institute of Biochemistry, Martinsreid, Germany) [16]. Rabbit polyclonal antibodies to entactin/nidogen [17], laminin α5 [4], laminin α4 [18], and laminin γ2 [19] were produced as described. Antibody against integrin α3 was from C. Michael DiPersio (Albany Medical College). Antibody against BP180 (also known as bullous pemphigoid BP antigen 2 and type XVII collagen, a major component of the epidermal anchoring complex) was from Zhi Liu (University of North Carolina, Chapel Hill, NC). Antibodies purchased from commercial suppliers were anti laminin α2 (4H8-2, Alexis Biochemicals, San Diego, CA), PECAM, integrin β4 (Pharmingen, San Diego, CA), laminin γ1, laminin β1, integrin α6, aquaporin-5, CC26, pro-surfactant protein C (SP-C) (Chemicon, Temecula, CA), β-tubulin IV (Biogenex, San Ramon, CA), and α-smooth muscle actin (Sigma, St. Louis, MO). FITC- and TRITC-conjugated anti-rabbit and anti-rat secondary antibodies were from Jackson ImmunoResearch Laboratories (West Grove, PA). FITC-anti-mouse IgG2b secondary antibody was from ICN Biomedicals, Inc. (Costa Mesa, CA).

### Immunofluorescence microscopy

Newborn pups were sacrificed by decapitation and immediately immersed in Tissue Tek OCT embedding medium (Sakura Finetek, Torrance, CA), frozen in liquid nitrogen-cooled 2-methylbutane, and sectioned at 6 μm on a cryostat. Sections were blocked with 10% normal goat serum in 1% BSA/PBS, then incubated with primary antibody diluted in 1% BSA/PBS. Slides were washed with PBS, and incubated with secondary antibody diluted in 1% BSA/PBS. Slides were again washed with PBS and mounted in Vectashield (Vector Laboratories, Temecula, CA). For laminin α4 staining, sections were fixed in 4% paraformaldehyde for 10 minutes, washed in PBS, treated with 0.1 M glycine, pH 3.5, for 10 minutes, washed in PBS, treated with 0.1% SDS at 55°C for 1 hour, washed in PBS, blocked, and stained as with other antibodies. For PECAM staining, sections were fixed in 100% ethanol prior to addition of the PECAM antibody. Antibody dilutions were 1:500 for laminins α1-α4 , 1:600 for laminin α5, 1:200 for laminin γ1, 1:800 for laminin γ2, and 1:50 for aquaporin-5.

### Electron microscopy

For transmission electron microscopy, lungs from newborn pups were fixed in 3% glutaraldehyde in 0.1 M sodium cacodylate buffer, postfixed with aqueous 1.25% osmium tetroxide, stained with 4% aqueous uranyl acetate, dehydrated through an ethanol series, embedded in Polybed, sectioned on a Reichert-Jung Ultra Cut, post-stained in 4% uranyl acetate and lead citrate, and viewed on a Zeiss 902 electron microscope. All reagents for electron microscopy were purchased from Electron Microscopy Sciences (Ft. Washington, PA) except Polybed (Polysciences, Warrington, PA).

### Histology and immunohistochemistry

Thoraces of newborn pups were isolated by decapitation and transection at the level of the liver. Thoraces were fixed in 4% paraformaldehyde in PBS, dehydrated in graded ethanols, embedded in paraffin, sectioned, and stained with hematoxylin and eosin for light microscopy.

For staining with β-tubulin, CC26, and SP-C antibodies 5 μm paraffin sections were immunostained using Vectastain Elite ABC and MOM staining kits (Vector Laboratories). Antigen unmasking prior to immunostaining with the SP-C antibody was performed using citrate buffer together with a pressure cooker (Biocare Medical, Carlsbad, CA) according to the manufacturer's instructions. Antibodies were developed with DAB (Vector Laboratories) and counterstained with hematoxylin. Antibody dilutions were 1:2500 for β-tubulin, 1:200 for CC26, and 1:5000 for SP-C.

### Lung bud culture

Lung rudiments dissected from E12.5 embryos were maintained for 72 h at 37°C on Transwell filters (Corning Costar, Cambridge, MA) in DMEM with 10% fetal bovine serum, 2 mM glutamine and 1% antibiotic/antimycotic [18]. Branching morphogenesis was quantified by counting terminal peripheral end buds at 0, 24, 48, and 72 h in culture.

### Morphometry

Chord length measurements for saccules were performed as described [20]. Briefly, ten serial slides at least 50 μm apart, which contained 5 μm sections of paraffin-embedded newborn *Lamc2-/- *and littermate control lungs (at least 3 of each) were stained with H&E. Random images were acquired with the 40× objective on a Nikon Optiphot II microscope and a Zeiss Axiocam digital camera. Chord length was determined using the NIH image program. Fields containing large airways and vessels were excluded. Statistical analysis was determined by two-tailed Student's t-Test.

## Results

### Histology of Lamc2-/- lungs

**Figure 1:**
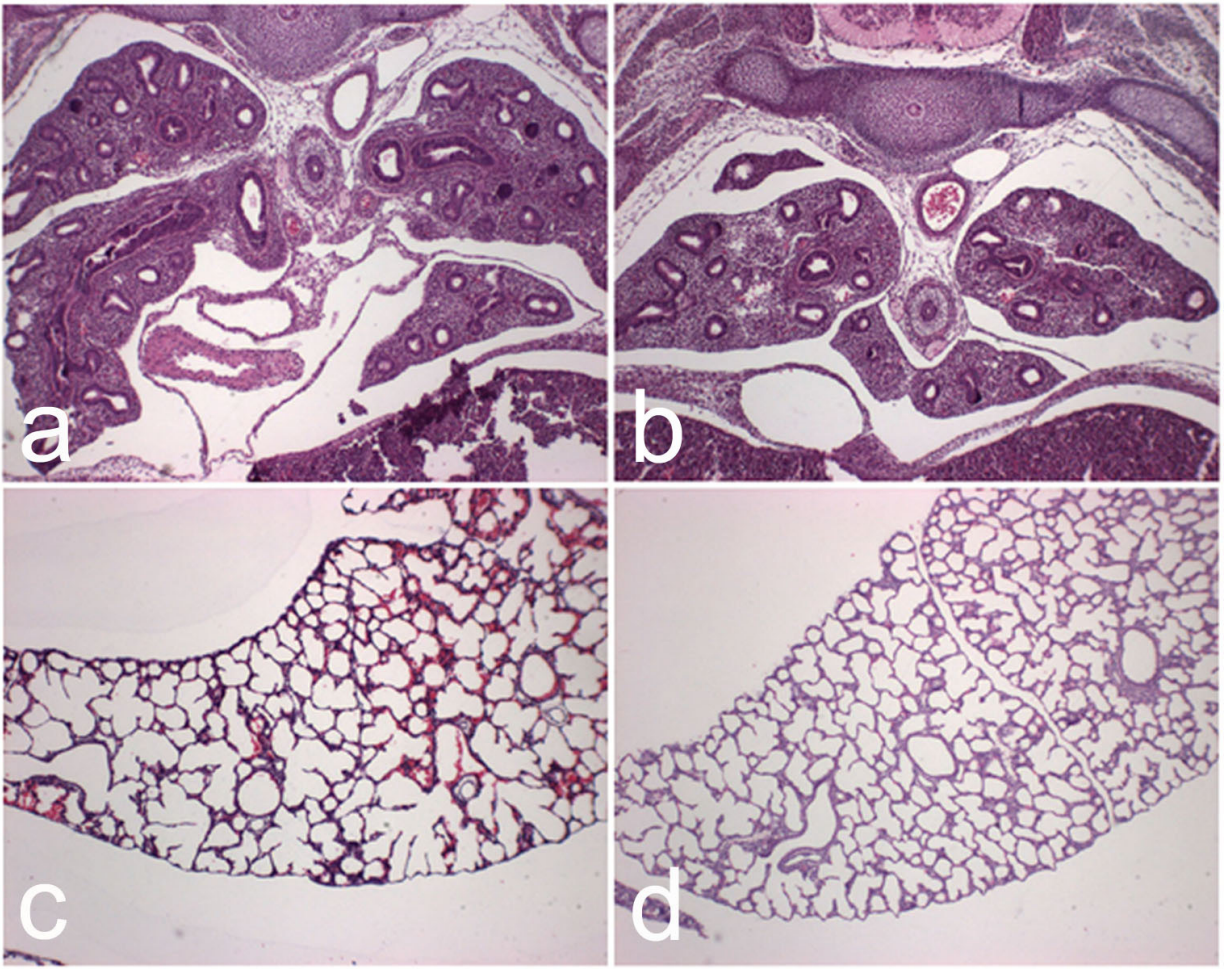
*Lung histology and morphometry*. Paraffin sections (5 μm) of *Lamc2-/- *(a, c) and littermate control (b, d) lungs at E14.5 (a, b) and P1-2 (c, d) were stained with hematoxylin and eosin. At E14.5, histology of *Lamc2-/*- (a) lung was indistinguishable from those of littermate control (b). At P1-2 *Lamc2-/- *(c) lungs had mildly enlarged saccules compared with those of littermate control (d). Original magnification 100× (a, b), and 40× (c, d).

*Lamc2-/- *mice die within 3–5 days of birth presumably due to failure to thrive and involvement of the oral and gastroesophageal mucosa [15]. Lungs of *Lamc2-/- *(Fig. 1a) and littermate control (Fig. 1b) mice did not show significant differences in structural morphology at E14.5. Comparison of post-natal day 1–2 lungs from *Lamc2-/- *(Fig. 1c) and littermate control (Fig. 1d) showed an increase in saccular size. Measurement of chord length confirmed the increase in the *Lamc2-/- *(25 μm) as compared to littermate control (20 μm) saccule size, however this difference was not statistically significant (p < 0.07, Student's t-Test).

### Basement membrane composition and ultrastructure

**Figure 2:**
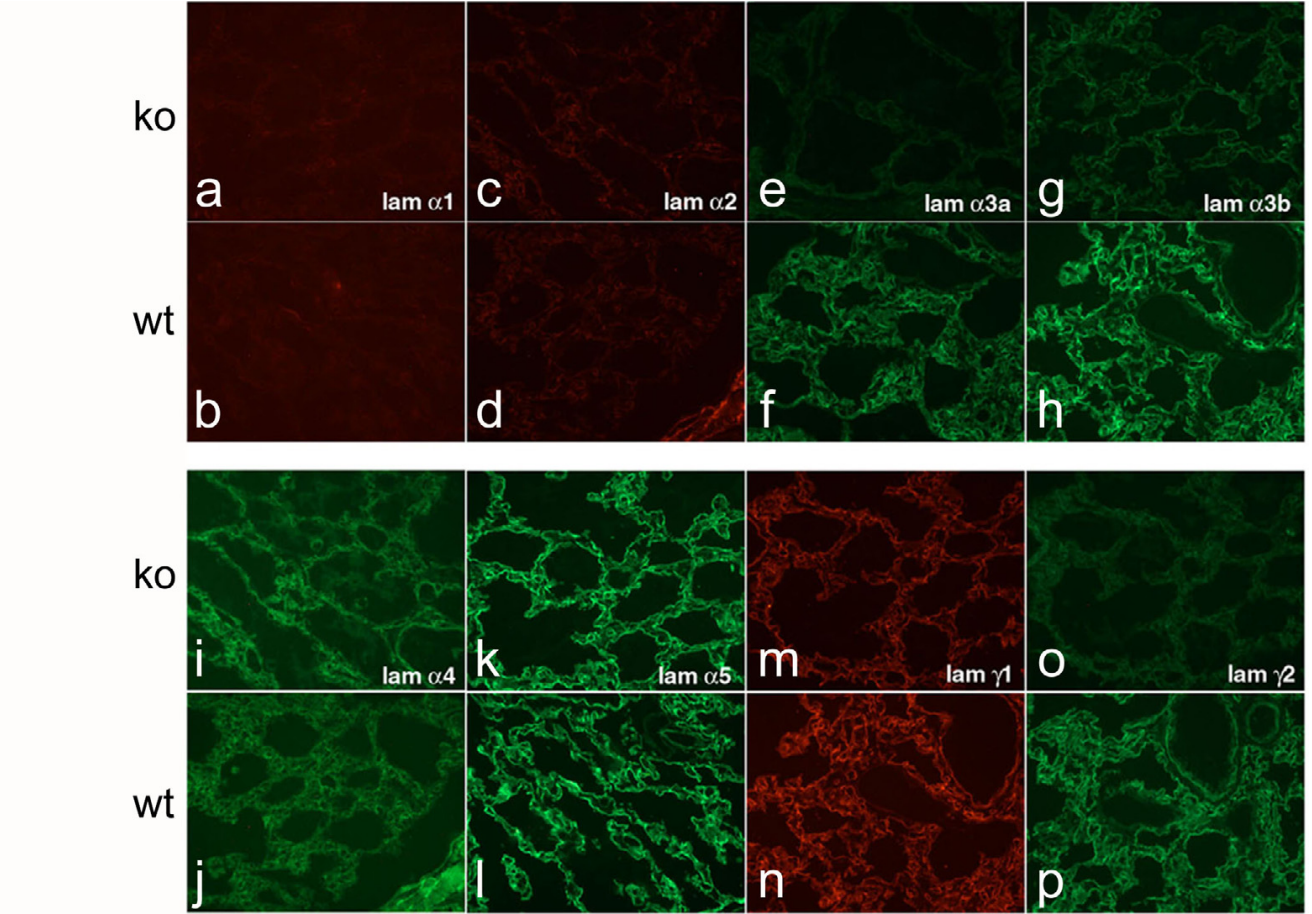
*Deposition of laminin chains*. Sections from newborn littermate control (b, d, f, h, j, l, n, p) and *Lamc2-/- *lungs (a, c, e, g, i, k, m, o) were stained with antibodies to laminins α1–5 and γ1–2. The laminin α1 (a, b) and α2 (c, d) chains were not detected at this stage. Laminin α3A (e-f) and α3B (g-h) chains were present but diminished in the airway basement membrane of *Lamc2-/- *(e, g) compared to littermate control (f, h) lungs. Laminin α4 (i-j), α5 (k-l), and γ1 (m-n) chains were present and similar in *Lamc2-/- *(i, k, m) and littermate control (j, l, n) lungs. Laminin γ2 is absent in *Lamc2-/- *(o) compared with littermate control (p) lungs. Original magnification 200×.

Perturbation of laminin expression often leads to compensation by another laminin chain [21,22]. Previously, we found that deletion of laminin α5 expression by lung epithelial cells was associated with ectopic deposition of laminin α4 in airway basement membranes [11]. To determine if deficiency of laminin γ2 is associated with changes in appearance of other laminin chains, we examined the lungs of *Lamc2-/- *mice by immunofluorescence for laminin α1–5, β1, and γ1–2 chains. Targeted deletion of laminin γ2 was confirmed by lack of staining for laminin γ2 in the *Lamc2-/- *lungs (Fig 2o). In the absence of laminin γ2, expression of laminin α3A (Fig 2e) and α3B (Fig 2g) chains were markedly diminished but not absent and the expression of other laminin chains was similar to littermate controls (Fig 2). Since the laminin γ2 chain is found only in laminin-332, this result is expected and parallels the finding in the skin of *Lamc2-/- *mice [15]. Immunofluorescence staining was also performed with antibody against entactin/nidogen but no differences were noted between *Lamc2-/- *and littermate controls (data not shown).

**Figure 3:**
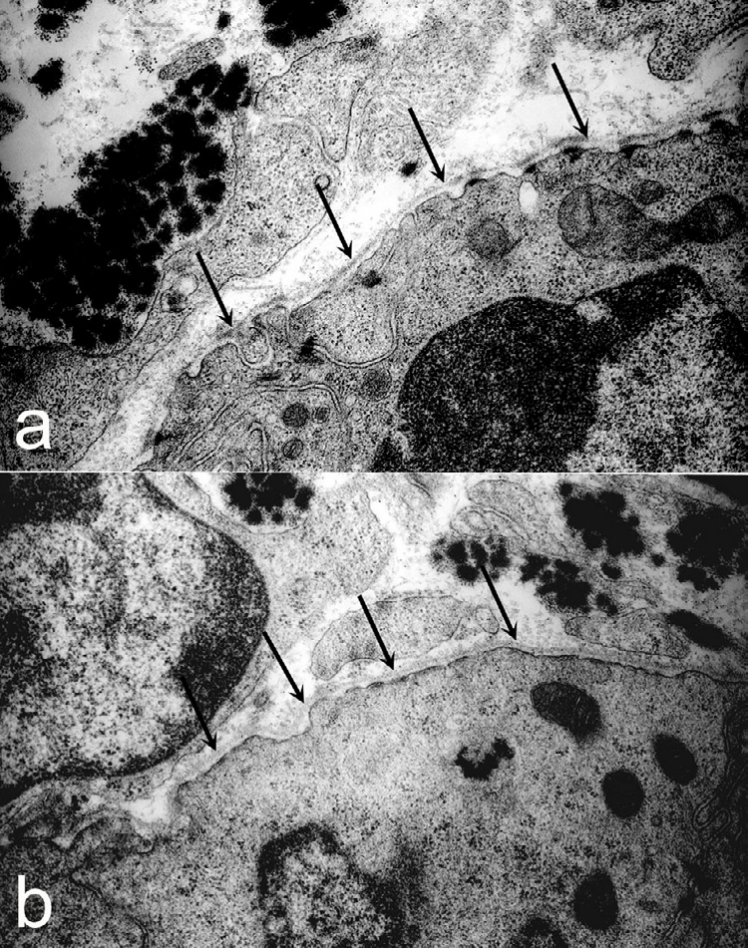
*Airway basement membrane ultrastructure*. Transmission electron micrographs of airways in both newborn littermate control (a) and *Lamc2-/- *(b) lungs revealed a continuous, electron dense basement membrane (arrows) underlying airway epithelial cells.

Ultrastructural analysis of conducting airway basement membrane in post-natal day 1 lung was accomplished by transmission electron microscopy. A continuous, well-defined, linear lamina densa of equivalent thickness was seen in both *Lamc2-/- *(Fig. 3b, arrows) and littermate control (Fig. 3a, arrows) airway basement membranes. Thus, although absence of laminin γ2 leads to decrease in components of laminin-332 it does not affect basement membrane formation or change its morphology.

### Lung branching morphogenesis

**Figure 4:**
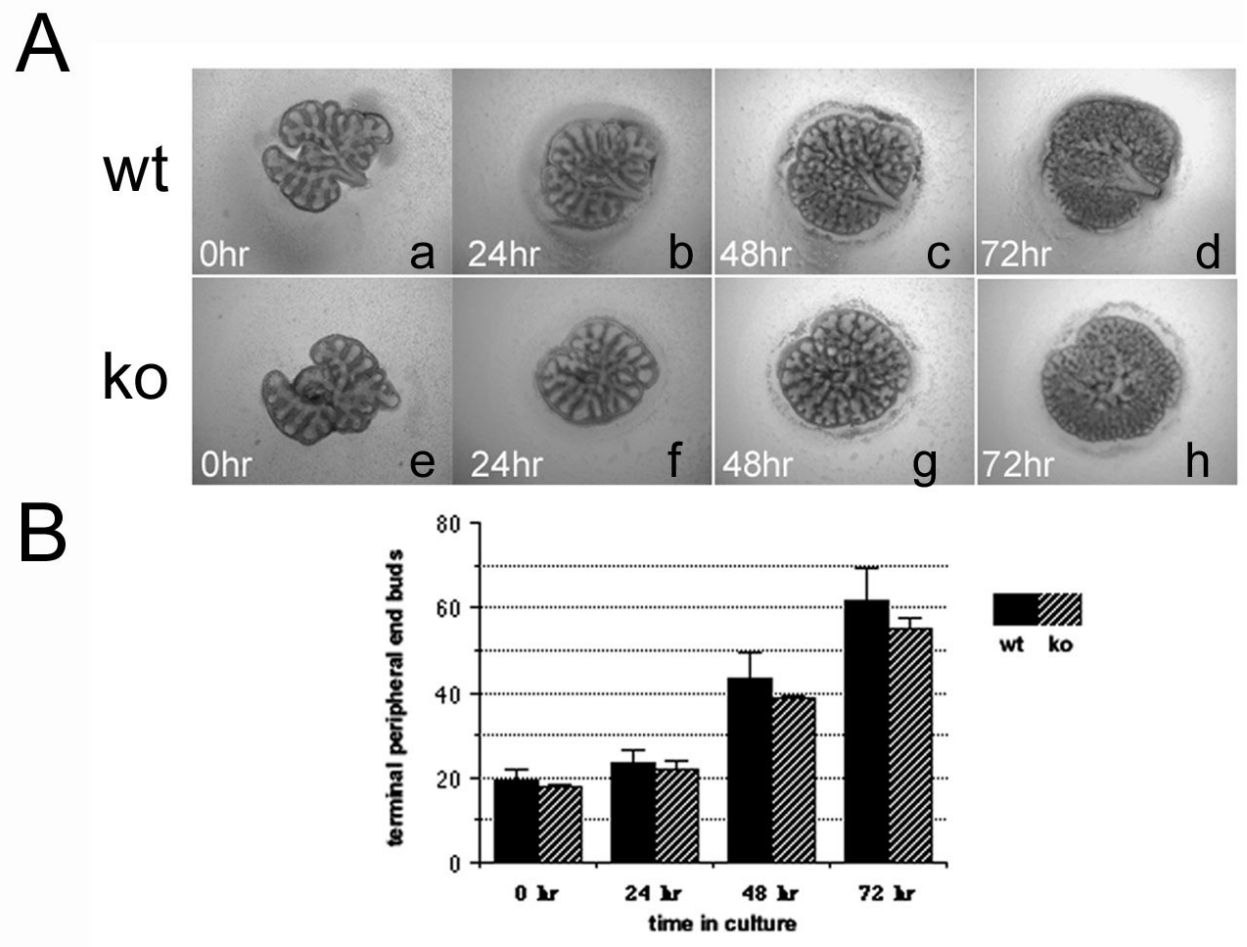
*In vitro branching morphogenesis*. Lung buds were removed from E12.5 littermate control and *Lamc2-/- *embryos and grown in culture for 72 hours. (A) Images of littermate control (a-d) and *Lamc2-/- *(e-h) lung buds were acquired at time of explant and after 24, 48, and 72 hours in culture. (B) Terminal peripheral airway buds were quantified and averaged (+/- SD) for each time point. No statistically significant differences were observed in littermate control and *Lamc2-/- *lungs. Analysis was performed with lung buds from 2 separate litters with at least 3 buds each for *Lamc2-/- *and littermate control. Original magnification 40×.

Because laminin γ2 has increased expression in branching clefts during the pseudoglandular stage of lung development, it has been hypothesized that laminin γ2 has a role in branching morphogenesis [14]. Accordingly, E12.5 littermate control (Fig. 4a–d) and *Lamc2-/- *(Fig. 4e–h) lung buds were removed and cultured for 3 days. The number of terminal peripheral end buds was quantified from photographs taken on day of explant and after 24, 48, and 72 h in culture (Fig. 4B). While the absolute number of terminal peripheral end buds was consistently less in *Lamc2-/- *lungs compared with littermate controls, this difference was not significant and the rate of increase in terminal peripheral end buds was similar in *Lamc2-/- *and littermate controls. From this, we conclude that laminin γ2, i.e. laminin-332, is not required for lung branching morphogenesis.

### Epithelial cell differentiation

**Figure 5:**
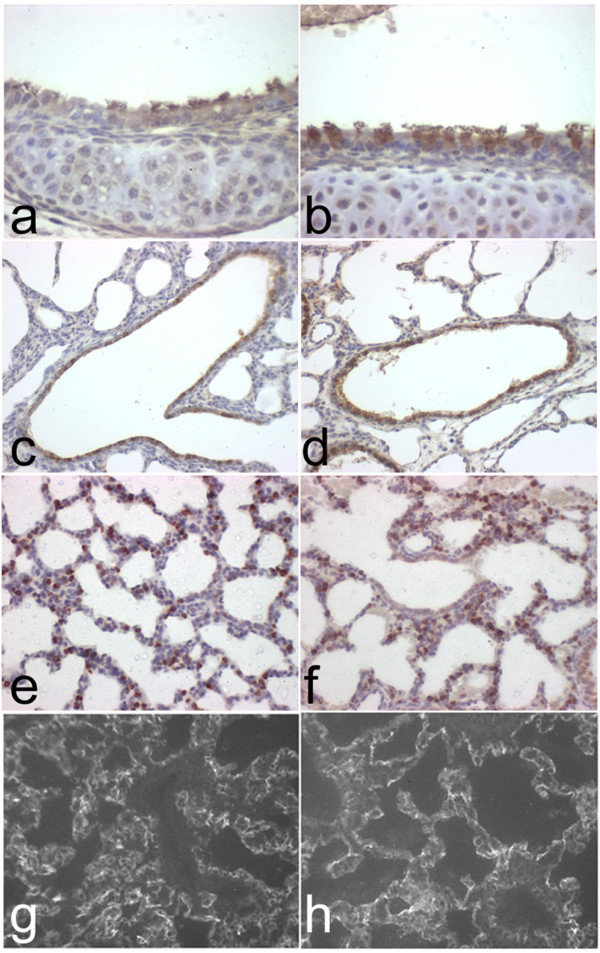
*Epithelial cell differentiation*. Sections of P1-2 littermate control (a, c, e, g) and *Lamc2-/- *(b, d, f, h) lungs were stained with antibodies to epithelial cell markers. Staining with the β-tubulin IV antibody for ciliated cells revealed similar staining in tracheas of littermate control (a) and *Lamc2-/- *(b) lungs. Staining with the CC26 antibody for Clara cells revealed similar findings in littermate control (c) and *Lamc2-/- *(d) lungs. Staining for the alveolar type II cell-specific marker pro-SP-C revealed no difference in proSP-C positive cells in littermate control (e) and *Lamc2-/- *(f) lungs. Immunofluorescence for the alveolar type I cell-specific marker aquaporin-5 revealed similar patterns in the *Lamc2-/-*lung (h) compared with littermate control (g). Original magnification 200×.

Absence of laminin α5 in the developing lung leads to abnormal differentiation of lung epithelial cells as shown by a marked decrease in alveolar type II cells and a near absence of alveolar type I cells [11]. As laminin γ2 co-localizes with laminin α5 in sub-epithelial basement membranes of developing lungs, we investigated whether lack of laminin γ2 would affect epithelial cell differentiation. Immunohistochemical staining against β-tubulin to detect ciliated cells (Fig. 5a–b), CC26 to detect Clara cells (Fig. 5c–d), PAS to detect mucous cells (data not shown), SP-C to detect alveolar type II cells (Fig. 5e–f), and aquaporin-5 to detect alveolar type I cells (Fig. 5g–h) all showed similar staining in *Lamc2-/- *(Fig. 5b, d, f, h) and littermate control (Fig. 5a, c, e, g) lungs. From these studies, we conclude that laminin γ2 is not required for lung epithelial cell differentiation.

### Endothelial and smooth muscle cell development

Although no differences in epithelial differentiation were noted, we examined development of other cell compartments. Endothelial cell and smooth muscle cell development were assessed with immunofluorescence staining for PECAM and α-smooth muscle actin, respectively. As with the epithelial cell markers, no differences in staining patterns or intensity were detected using either the endothelial cell marker or the smooth muscle cell marker (data not shown).

### Hemidesmosomes

**Figure 6:**
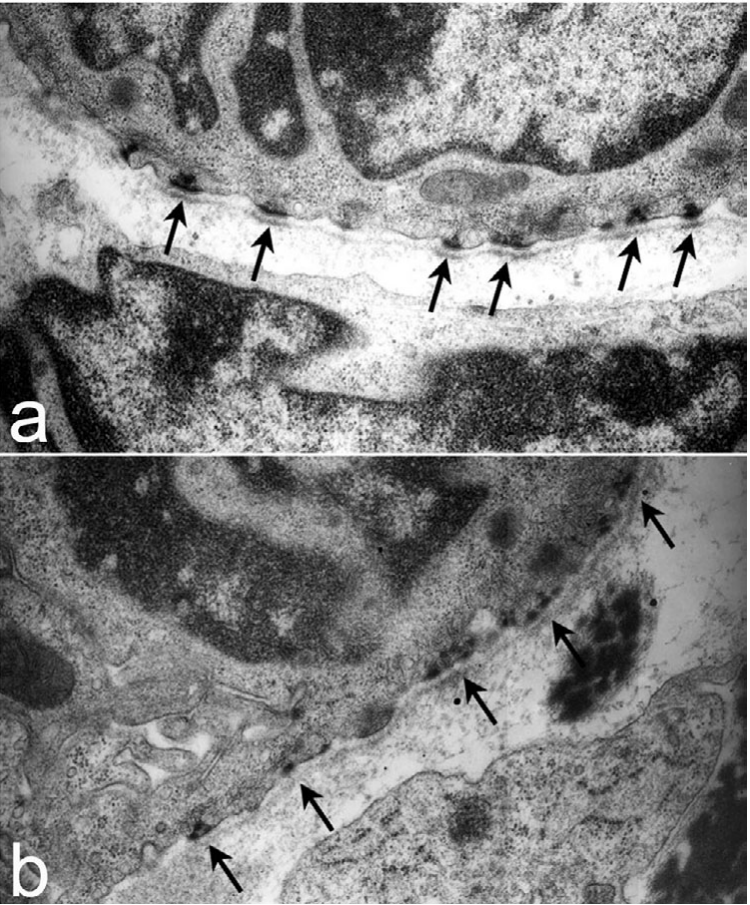
*Ultrastructure of tracheal hemidesmosomes*. Tracheas from P1-2 *Lamc2-/- *and littermate control mice were processed for transmission electron microscopy. Littermate control tracheas (a) had well-defined, organized hemidesmosomes with darkened areas in the lamina densa abutting the hemidesmosome (arrows). In contrast, hemidesmosomes in *Lamc2-/- *tracheas (b) were less organized, the intracellular component was more diffuse, and the lamina densa directly below the hemidesmosomal areas lacked the electron density seen in the littermate control (arrows).

**Figure 7:**
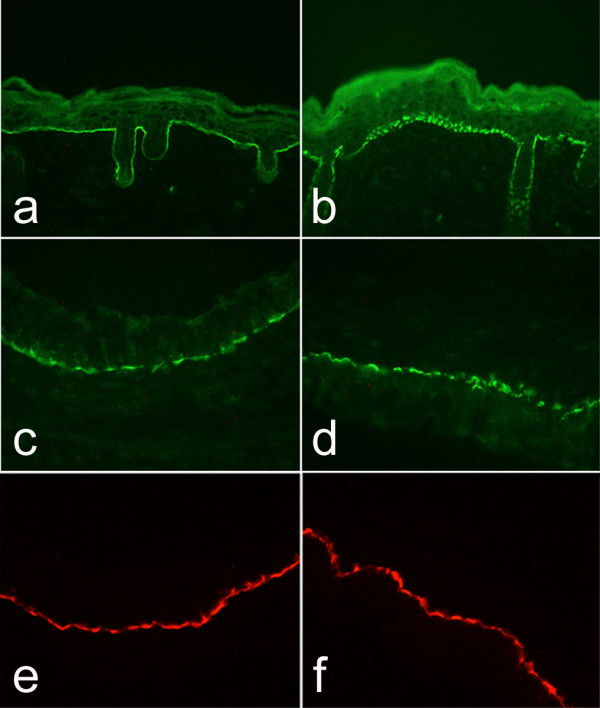
*Hemidesmosomal proteins*. Sections from P1-2 littermate control and *Lamc2-/- *skin and tracheas were stained with antibodies against BP180 and integrin β4 to examine the distribution of hemidesmosomal proteins. In the skin (a-b), staining with BP180 antibody showed a continuous pattern in the littermate control mouse, whereas a discontinuous pattern was seen with the *Lamc2-/- *(b) mouse. In the trachea (c-d), BP180 staining was similar in littermate control (c) and *Lamc2-/- *(d) mice. Immunofluorescence with antibody against integrin β4 also did not reveal a difference in littermate control (e) and *Lamc2-/- *(f) tracheas. Original magnification 200×.

Laminin-332, the only laminin to contain laminin γ2, is the only laminin associated with hemidesmosomes, a specialized transmembrane cell/matrix adhesion structure found at the basal aspect of basal cells of squamous and transitional epithelia. Hemidesmosomes are essential for basement membrane zone integrity with disruption leading to epidermolysis bullosa, a group of heritable blistering diseases [23]. In the lung, hemidesmosomes are restricted to tracheal and bronchial epithelial cells [24]. Since laminin-332 is required for hemidesmosome formation, we examined tracheal hemidesmosomes of *Lamc2-/- *mice. By transmission electron microscopy of post-natal day 1 tracheal epithelial cells, we found that hemidesmosomes of *Lamc2-/- *(Fig. 6b, arrows) mice are different than those of littermate controls (Fig. 6a, arrows). As seen in the cutaneous hemidesmosomes of *Lamc2-/- *mice [15], tracheal hemidesmosomes are poorly formed with disorganization of the inner and outer plaques and decreased electron density of the lamina densa of the basement membrane underlying the hemidesmosome. Immunofluorescence staining for BP180 and integrin β4, additional components of the hemidesmosome, in tracheal epithelium did not reveal a difference between littermate control (Fig. 7c, 7e, respectively) and *Lamc2-/- *(Fig 7d, 7f, respectively) lungs. This is in contrast to what was found in *Lamc2-/- *skin (Fig. 7b) where immunostaining for all hemidesmosomal proteins was diffuse and punctate while the littermate control skin (Fig. 7a) was continuous and localized [15].

### Laminin-322 receptors

**Figure 8:**
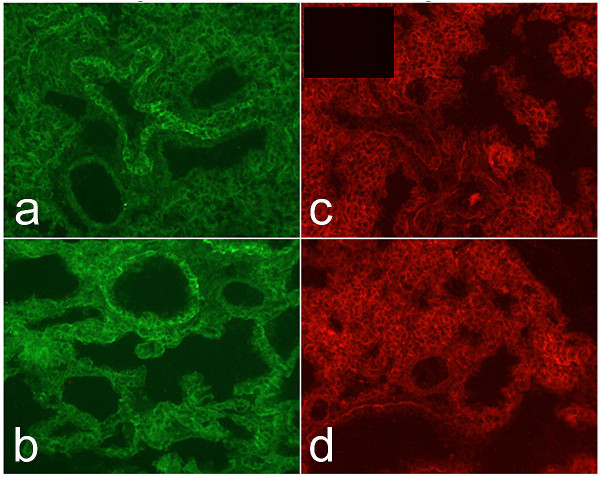
*Expression of laminin-332 receptors*. Sections from P1-2 littermate control and *Lamc2-/-*lungs were stained with antibodies against integrins α3 and α6. No differences in intensity or localization were detected by immunofluorescence for integrins α3 (a-b) or α6 (c-d) between littermate control (a, c) and *Lamc2-/- *(b, d) lungs. No immunofluorescence was detected when primary antibody was omitted from the procedure (inset panel c). Original magnification 200×.

Manipulation of laminin ligands often results in perturbation of its receptors. Deletion of the laminin α5 chain leads to abnormal localization of its cellular receptors Lutheran and integrin α3 [11,25]. Accordingly, we examined the localization of two common laminin-332 receptors, integrin α3 and integrin α6. Integrin α3 is important for cell-adhesion and integrin α6 pairs with integrin β4 in the hemidesmosome. Immunofluorescence with antibodies against integrins α3 (Fig 8a–b) and α6 (Fig 8c–d) showed similar staining in littermate control (Fig 8a, c) and *Lamc2-/- *(Fig. 8b, d) lungs.

## Discussion

Laminin-332, the only laminin containing the laminin β3 and γ2 chains, was first detected in human basement membranes and underneath hemidesmosomes decades ago [26]. The primary structures of the individual chains of laminin-332 were determined in the early 1990s and laminin-332 and its components have been studied extensively in subsequent years. Laminin-332 containing the laminin γ2 chain is produced by epithelial cells and is widely distributed in basement membranes of most epithelia, including skin, lung, gastrointestinal tract, kidney, prostate, ovary, and blood vessels of spleen and thymus [12,27,28]. Knockout and transgenic mice technologies have enabled exploration of specific functions of individual laminin-332 components in mouse development. Targeted deletion of the laminin α3 chain leads to perinatal death with a severe blistering disease similar to human junctional epidermolysis bullosa, and defective late stage differentiation of ameloblasts in developing incisors [29]. A null mutation in the *Lamb3 *gene from spontaneous insertion of an intracisternal-A particle at an exon/intron junction also results in blisters and death hours after birth [30]. *Lamc2-/- *mice suffer perinatal death and exhibit skin lesions that recapitulate human junctional epidermolysis bullosa with induced apoptosis in the basal cells of the abnormal skin [15]. Although laminin-332 is seen in many organs, most of the focus has been on skin during the characterization of mice with mutations in any of the laminin-332 constituents. In this report, we focus on lung development in *Lamc2-/- *mice.

*Lamc2*, present in laminin-332, is found in the epithelial airway and alveolar basement membranes of adult lungs and epithelial basement membranes of lung from the pseudoglandular to the alveolar stage of lung development [14]. In human lung during the pseudoglandular stage, immunodetection of laminin γ2 and laminin-332 revealed higher intensity of fluorescence in the clefts of the ramifications of the growing respiratory tubules leading the authors to hypothesize a role in branching morphogenesis [14]. Because laminin-332 co-localizes with laminin-111, which is a known effector of lung branching morphogenesis in vitro, speculation of a role for laminin-332 in branching morphogenesis was plausible. In our study, we were able to directly examine branching morphogenesis in the absence of laminin γ2 and laminin-332 through use of the *Lamc2-/- *mouse. We found that deficiency of laminin γ2 did not affect lung branching morphogenesis of in vitro lung bud cultures. This result is reminiscent of our finding normal lung branching morphogenesis in the *Lama5-/- *mouse, even though laminin α5 co-localized with laminin α1 [18]. Of note, the null mutation of integrin α3, a major ligand for both laminin α3 and laminin α5 containing laminins, led to abnormal branching morphogenesis [31] thus normal branching morphogenesis in laminin 332 deficient and laminin α5 null mice is rather unexpected. In the case of the *Lamc2-/- *mouse, only laminin-332 is absent so that other laminin α3 chain containing laminins (laminins-311 or -321) are still present and can contribute to the process of branching morphogenesis. An alternate, and perhaps more attractive, conclusion is that within the epithelial-derived laminin chains (those with laminin α1, α3, and α5 chains), no redundancy of function exists for laminin chains and only laminins containing the laminin α1 chain exert effects on lung branching morphogenesis. To resolve this, one needs to examine lung branching morphogenesis in a *Lama1-/- *mouse or a double *Lama3/Lama5 *knockout mouse.

Again, based on localization of laminin-332 and γ2 during development, roles in lung epithelial differentiation and alveolization were also suggested [12,14]. This idea fits with studies showing that laminin-332 stabilizes the phenotype of primary alveolar epithelial cells in culture [32-34]. However, we found normal expression and localization of markers of airway and alveolar epithelial cells in *Lamc2-/- *lungs. This finding contrasts with the lungs of mice lacking laminin α5 in which there is a marked impairment in differentiation of distal epithelial cells [11]. With respect to lung alveolization, we did note a mild increase in saccule size in the *Lamc2-/- *compared with littermate controls. Whether laminin γ2 or laminin-332 is important for later stages of lung development, specifically alveolization remains to be determined since *Lamc2-/- *mice died before alveolization occurs. That lack of laminin γ2 did not significantly affect lung epithelial cell differentiation while perturbation of laminin α5 had a dramatic effect again indicates that laminins have specific, non-overlapping functions during lung development.

In the absence of a laminin chain, compensation by ectopic expression of another laminin of the same chain group can occur. *Lamb1 *compensates for lack of *Lamb2 *in the kidney, upregulation of *Lama4 *is seen with loss of *Lama2 *in muscle, deletion of *Lama5 *leads to ectopic *Lama2 *and *Lama4 *in ectoderm and intestines [21,22,35,36]. However, a compensatory response was not detected with deletion of laminin γ2 in the lung or in the skin. The reason for this is unknown but it may relate to the uniqueness of laminin-332 in that it is the only laminin known to contain the β3 and γ2 chains and it is the only laminin present in hemidesmosomes.

By transmission electron microscopy, tracheal hemidesmosomes in *Lamc2-/- *mice were different from those of the littermate control. This finding is consistent with cutaneous hemidesmosomes in the *Lamc2-/- *and the *Lama3-/- *mice. However, even though the hemidesmosomes appeared abnormal at the ultrastructural level, immunofluorescence staining for other components of hemidesmosomes was similar between *Lamc2-/- *and littermate control lungs. In contrast, immunostaining for cutaneous basement membrane zone proteins in *Lamc2-/- *and *Lama3-/- *both showed abnormal distribution of these proteins compared with controls [15,29]. In addition, skin epithelial and oral and bladder mucosa of *Lamc2-/- *and *Lama3-/- *had areas of blister formation while no areas of blistered epithelium were found in *Lamc2-/- *tracheas. This suggests that abnormal hemidesmosomes in *Lamc2-/- *tracheas did not produce a functional defect or that the tracheas are not mechanically stressed enough to blister. Alternatively, tracheal hemidesmosomes may have different function compared to hemidesmosomes in other tissues. In people with epidermolysis bullosa, the main pathologic feature is skin blistering with abnormal hemidesmosomes. Rare cases of laryngotracheal involvement have been reported but airway obstruction has not been implicated as a significant cause of mortality in these patients [37-39]. Thus, our finding of normal appearance, integrity, and presumably function, of tracheal epithelium despite abnormal hemidesmosomes in the *Lamc2-/- *mice is consistent with infrequent abnormalities in humans.

While we did not observe a significant role for laminin γ2 and laminin-332 in lung development, physiologic roles of this laminin must exist. Laminin-332 may facilitate alveolar epithelial repair via effects on cell migration. By in situ hybridization, immunohistochemistry, and immunoelectron microscopy, regenerating epithelial cells in cryptogenic organizing pneumonia and in idiopathic pulmonary fibrosis both express laminin γ2 in response to injury [40]. In addition, a recent report shows that laminin γ2 is not only present in the basement membrane but also in the cytoplasm of injured epithelial cells and in columnar epithelium of allergic asthmatics [41]. Moreover, this laminin may influence tumor cell behavior [42]. Tumor cell lines often express laminin-332 and the expression is enhanced by epidermal growth factor [43]. In lung tumors, expression of the laminin γ2 chain was strong in squamous cell carcinomas, adenocarcinomas, and large cell carcinomas, with immunoreactive cells localizing to the epithelial-stromal interface of tumor clusters [44]. Inactivation of laminin-322 genes by aberrant methylation in prostate cancer and bladder cancer samples correlated with poor prognosis [45-47]. Laminin γ2 can be found in the cytoplasm of carcinoma cells invading into interstitial stroma while laminin α3 and β3 chains are only found in the basement membrane [48].

## Conclusion

In summary, analysis of *Lamc2-/- *lungs reveals that laminin γ2 and its associated laminin-332 are not essential for virtually normal lung development to the saccular stage. Peri-natal death of *Lamc2-/- *mice prior to completion of alveolization precludes a definitive conclusion about the requirement of *Lamc2 *for alveolization. However, the prominence of laminin γ2 in alveolar walls during lung development and the adult lung points to important physiologic functions.

## Competing interests

The author(s) declare that they have no competing interests.

## Authors' contributions

NN participated in the design of the study, carried out the lung bud cultures, immunofluorescence staining, statistical analysis, supervised the optimization of immunostaining, evaluated the data, and drafted the manuscript. JS carried out the immunohistochemical staining, morphometry, evaluated the data, and helped to draft the manuscript. LP and JU produced *the Lamc2-/- *mouse and helped to draft the manuscript. GM made the laminin γ2 antibody and helped to draft the manuscript. RS conceived the study, participated in its design and coordination, evaluated the data, and helped to draft the manuscript. All authors read and approved the final manuscript.
